# Using Design of Experiments Methods for Assessing Peak Contact Pressure to Material Properties of Soft Tissue in Human Knee

**DOI:** 10.1155/2013/891759

**Published:** 2013-09-08

**Authors:** Marjan Bahraminasab, Ali Jahan, Barkawi Sahari, Manohar Arumugam, Mahmoud Shamsborhan, Mohd Roshdi Hassan

**Affiliations:** ^1^Department of Mechanical and Manufacturing Engineering, Universiti Putra Malaysia, 43400 Selangor, Malaysia; ^2^Faculty of Engineering, Semnan Branch, Islamic Azad University, Semnan, Iran; ^3^Institute of Advanced Technology, ITMA, Universiti Putra Malaysia, Selangor, Malaysia; ^4^Department of Orthopedic Surgery, Faculty of Medicine & Health Science, Universiti Putra Malaysia, Selangor, Malaysia; ^5^Department of Mechanical Engineering, K.N. Toosi University of Technology, Tehran, Iran

## Abstract

Contact pressure in the knee joint is a key element in the mechanisms of knee pain and osteoarthritis. Assessing the contact pressure in tibiofemoral joint is a challenging mechanical problem due to uncertainty in material properties. In this study, a sensitivity analysis of tibiofemoral peak contact pressure to the material properties of the soft tissue was carried out through fractional factorial and Box-Behnken designs. The cartilage was modeled as linear elastic material, and in addition to its elastic modulus, interaction effects of soft tissue material properties were added compared to previous research. The results indicated that elastic modulus of the cartilage is the most effective factor. Interaction effects of axial/radial modulus with elastic modulus of cartilage, circumferential and axial/radial moduli of meniscus were other influential factors. Furthermore this study showed how design of experiment methods can help designers to reduce the number of finite element analyses and to better interpret the results.

## 1. Introduction

Knee joint contact pressure is of critical importance in the mechanisms of knee pain and osteoarthritis [1, 2]. Computational models and finite element analyses (FEA) have been utilized to study contact characteristics of normal and injured knees, as well as total knee replacements (TKR) [3–8]. The purpose of these studies was to determine peak contact pressure in order to predict either tissue degradation of the knee or wear of ultra-high molecular weight polyethylene (UHMWPE) in TKR. Some biomechanical factors, such as material properties and geometries of tissues [9, 10], and knee kinematic [11] can affect the contact behavior of the knee and consequently the design of TKR. Impacts of horn attachments stiffness and meniscal material properties on tibiofemoral contact pressure using “semiautomatic” optimization method were investigated by Haut Donahue et al. [9], who set tolerances on the variables to restore the contact pressure to within a specified error. The authors, however, performed more than 60 analyses to determine whether an individual factor is of importance. Meanwhile, interaction effects between different factors were not considered in their study. In order to better interpret the effects of variations in the material properties of soft tissue, a powerful statistical approach is required to design computational experiments.

Design of experiments (DOE) is a formal mathematical method that helps to solve complicated problems and to save time and resources (cost) by reducing the number of required experiments (runs) while obtaining all the necessary information. However, reducing runs associate with decrease in resolution. Usually in an experiment, one or more factors are deliberately changed in order to observe the effect of these changes on one or more response variables. The statistical design of experiments is an efficient method for planning experiments so that it can be analyzed to yield valid and objective conclusions that can be obtained for a given amount of experimental efforts. Recently, there has been an increasing interest in use of DOE for sensitivity analysis based on FEA in biomedical applications [11–15]. So far, this method has only been applied in few studies related to the human knee joint [11, 14, 15]. Yao et al. [15] focused on the medial compartment of the knee and investigated the sensitivities of medial meniscal motion and deformation to material properties of soft tissues. They used Taguchi approach and central composite design to fit the finite element model (FEM) to the experimental data in the anterior cruciate ligament-deficient knee. Furthermore, Julkunen et al. [14] used a three-level fractional factorial design in combination with composition-based finite element model to determine the effect of different cartilage constituents on the mechanical response of the tissue. Due to the uncertainty in material properties [16], finite element analysis of the tibiofemoral joint becomes a very challenging mechanical problem. Therefore, the aims of this paper are to explore the most important parameters related to the material properties of meniscus and cartilage affecting the tibiofemoral joint contact pressure and to make a regression model based on main interaction and quadratic effects of variables to understand how they influence and minimize the error of FEA output. In this regard, fractional factorial design was applied in screening step and Box-Behnken method was used in response to surface method and optimization process.

## 2. Methods

### 2.1. Creation of Finite Element Analysis

**Figure 1:**
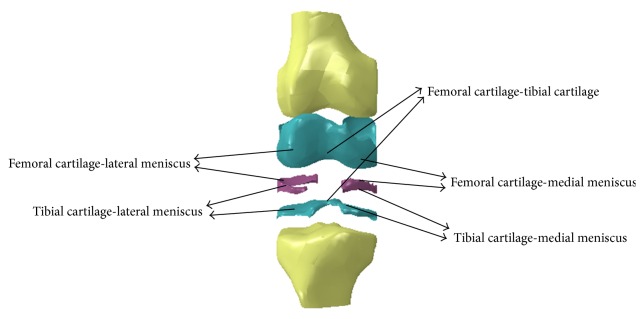
Different parts of FE model and contact pairs.

Geometries of bony structures and soft tissues were taken from a healthy human knee of a 24-year-old man. Solid models of the femur and tibia and geometries of soft tissues, including articular cartilages and menisci, were developed from the magnetic resonance images (MRI). Each image was taken at 3.2 mm interval in a sagittal plane. The obtained data, subsequently, was used to create a three dimensional computer aided design (3D CAD) model in order to import into ABAQUS 6.8 software (Dassault Systèmes Simulia Corp., Providence, RI, USA). The model consisted of two bony structures (femur and tibia), both the femoral and tibial articular cartilages, and both the medial and lateral menisci.  Figure 1 shows the generated 3D model in details. The model did not include ligaments. The finite element mesh generation was performed leading to 41709 linear 4-noded tetrahedron elements for articular cartilage and menisci (25293 for femoral cartilage, 9130 for tibial cartilage, 3866 for medial meniscus, and 3420 for lateral meniscus). Contact was defined between the femoral cartilage and meniscus, the meniscus and tibial cartilage, and femoral cartilage and tibial cartilage for both lateral and medial compartments, resulting in six contact-surface pairs. Completely general contact condition involving small sliding of pairs was applied on the model and all contact surfaces were modeled as frictionless. The cartilage in the knee is a complex structure, composed mainly of networks of collagen fibrils that embed water and a non-fibrillar matrix. The cartilage is known to be inhomogeneous and anisotropic material, but considering that the loading time of interest is related to a single leg stance and that the viscoelastic time constant for cartilage is approximately 1500 seconds from biphasic theory [3, 9], the elastic solution does not diverge from the biphasic solution [17]. The cartilage, therefore, was assumed to behave as a homogeneous linearly isotropic elastic material for contact pressure computations, similar to the previous studies [18, 19]. The meniscus, also, has similar structure to that of cartilage and it is also known to be inhomogeneous and anisotropic material, but various material property definitions can be found in the literature for this component [20–22]. Furthermore, the meniscus has a time constant, as large as 3300 seconds [9], and can also be considered as an elastic material for compression of the joint during the short loading times (single leg stance). In this study, the menisci were treated as linearly elastic, transversely isotropic material to represent the circumferential fiber arrangement. Femur and tibia were represented by rigid bodies because this is time efficient in a nonlinear analysis and accurate due to their much larger stiffness compared to that of soft tissues. Meanwhile, the previous study [3] confirmed that this simplification has no substantive effect on contact variables. Horn attachments, in the current model, were defined by 10 linear springs. For boundary conditions, the tibia was fully constrained and femur was constrained from rotation and free to translate in anterior-posterior, medial-lateral, and inferior-superior axes.

**Figure 2:**
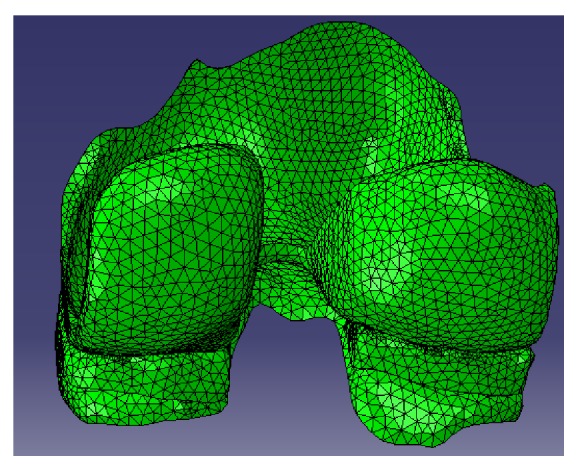
The finite element representation of the joint.

For validation of the model, static loads equivalent to 0, 500, 734, 800, 1000, 1500, 2000, and 2500 N were applied on the model at 0° flexion angle in order to compare with previously reported measurements and predictions [3, 29–31]. In this regard, the initial cartilage elastic modulus and Poisson's ratio were considered as 15 MPa and 0.475, respectively [25], and for menisci the primary values were moduli of 20 MPa in axial/radial directions and 140 MPa in circumferential direction. The values used for in-plane and out-of-plane Poisson's ratios and shear modulus were 0.2, 0.3, and 50 MPa, respectively [20, 21, 23, 24]. The stiffness of horn attachments was considered 200 N/mm, which resulted in 2000 N/mm total stiffness.  Figure 2 shows the finite element representation of the joint.

### 2.2. Verifying the Results of FEA

**Figure 3:**
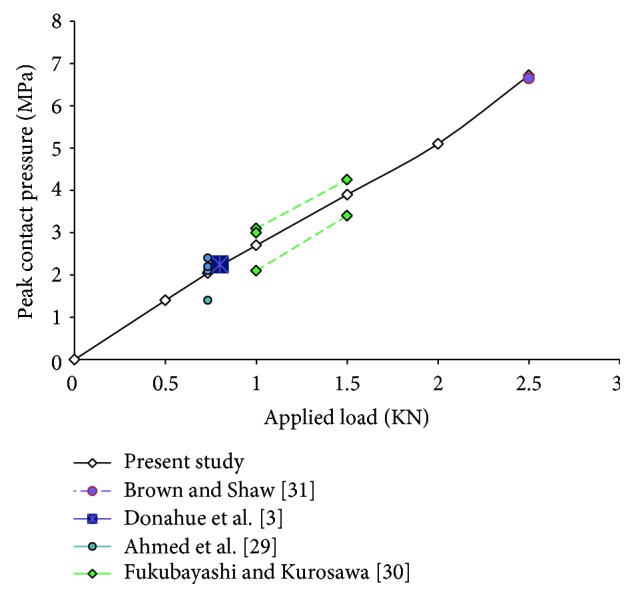
Comparison of the results of peak contact pressure on the tibial plateau.

The results of peak contact pressure for different magnitudes of force are shown in Figure 3. The applied force is transferred through the femur-meniscus, femur-tibia, and meniscus-tibia at the contact regions. The stresses were computed and it was seen that the total stress multiples by area equilibrate the total load in the knee joint. The predicted reaction forces at each loading condition, also, were in equilibrium with the applied load. Although, the finite element solution may have satisfied the equilibrium, indicating that the finite element solution was accurate to some extent, confidence in the validity of the model itself were obtained by comparing the computed values of the peak contact pressure with the previously reported measurements and predictions. Among the various researches that have measured the peak contact pressure on the tibial plateau [3, 4, 8–10, 29–31], studies of Brown and Shaw [31], Ahmed et al. [29], Fukubayashi and Kurosawa [30] and Donahue et al. [3] were chosen because they used a load application system with various compressive loads (734, 800, 1000, 1500, and 2500 N) at 0 flexion angle and computed the peak contact pressure on the tibial cartilage. It can be seen that, the results of present study fall well within the ranges provided by the literature. Hence the present results are verified. 

### 2.3. Design of Experiments

DOE starts with determining the objectives of an experiment. These objectives are as follows: comparative, screening, and modeling [32–34]. Objective of comparative designs is to find a suitable method for an initial comparison. Screening designs identify which factors are important and help to screen out unimportant factors. Response surface modeling seeks for one or more of the following objectives: hit a target, maximize or minimize a response or make it robust.

In this research, seven factors including axial/radial and circumferential elastic moduli of meniscus (*E*_2,3_ and *E*_1_), stiffness of meniscus horn attachment (*K*), in-plane and out-of-plane Poisson's ratios (*υ*_12_, *υ*_23_), shear modulus (*G*_12_), and elastic modulus of cartilage (*E*) were considered as initial variables for sensitivity analysis. Due to the large number of factors and levels, in the first step fractional factorial design was applied to screen out less significant factors. It is useful and efficient when full factorial design becomes unpractical [35]. Two levels for each factor impose (2^7^ = 128) treatment combinations for full factorial design, but the 1/8 fractional factorial design suggested 16, so it can be used as a rational way for choosing the treatment combinations of experiments. The 1/8 fractional design corresponds to resolution IV in which the main effects are not confounded with two-way interactions. However, a limitation of fractional factorial design is the use of only two levels for each factor and the responses are assumed to be approximately linear over the range of the factor levels chosen. More detailed discussions can be found in Montgomery [36]. In the next step, after screening out the less significant factors, Box-Behnken design was applied to do more investigations and hit the value of experiment in the FE model. The Box-Behnken is a good design in response surface methodology due to estimation of the parameters in the quadratic model. Furthermore, it is slightly more efficient than the central composite design [37], which was used by Yao et al. [15] in the sensitivity analysis of the knee joint. 

 In this study, sensitivity analysis was performed under 2500 (N) static load at full extension and the reference value of the peak contact pressure was taken from the experimental work of Brown and Shaw [31], which was equivalent to 6.5 (MPa). At this load, the optimum values of parameters were obtained by the estimated model based on Box-Behnken design. The predicted optimum values were subsequently tested for other applied loads at 0 flexion angle. The study of Brown and Shaw [31] was chosen because it measured the peak contact pressure on tibial cartilage under the same loading condition (static load of 2500 N at 0 flexion angle). The considered factors and their investigated ranges based on the literature are demonstrated in Table 1. The combination of parameters was generated and analyzed using Minitab software [38], and the design generators were E = ABC, F = BCD, and G = ACD.

## 3. Result and Discussion

### 3.1. Screening Analysis

Table 2 shows the treatment combinations and the results of peak contact pressure, according to fractional factorial design. The ranges in this step were chosen according to the most prevalent values used in the literature. For example, however the given range for elastic modulus of cartilage is 5–20 MPa, the majority of studies have considered values equal or more than 12 MPa [3, 4, 8, 9, 11, 39, 40]. 

**Figure 4:**
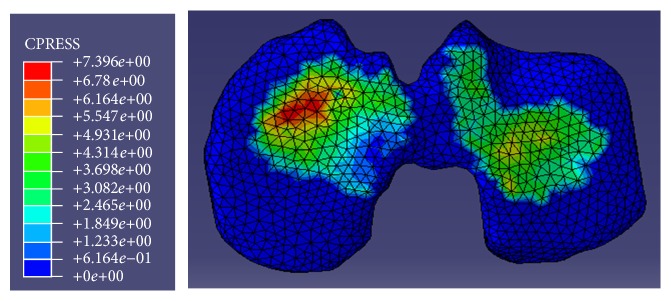
Contact pressure distribution in the tibial cartilage in first experiment.

The location of peak contact pressure was on the lateral compartment of the tibial cartilage in all FE analyses.  Figure 4 shows the distribution of contact pressure for the first experiment; the red region represents the maximum contact pressure. The maximum variations in the location of peak contact pressure were 0.10 mm in anterior-posterior direction to the anterior and 1.74 mm in medial-lateral direction to the lateral side.

Table 3 demonstrates both the magnitude and the importance of the parameters effects. Any absolute value of the effect greater than *α* = 0.05 is potentially important. Hence, factor E which represents the elastic modulus of cartilage has the most effect on peak contact pressure, followed by *E*_1_∗*K* (representing both circumferential and horn stiffness) and *E*_2,3_ (axial/radial modulus of meniscus), respectively. The other parameters are not significant at the 5% level. It can be seen that, however, other factors including *E*_1_ and *K* are not significant, interaction of *E*_1_ and *K* is significant. 

Interaction is the variation among the differences between means for different levels of one factor over different levels of the other factor; and since in resolution IV designs, two-factor interaction effects may be confounded with other two-factor interactions, screening out of factors should be done carefully, because confounding pattern makes it difficult to determine which factors are the most important ones. In this regard, Table 3 also shows alias structure up to order 3. The alias structure indicates which effects are confounded with each other. As shown in Table 3, effect of *E*_1_∗*K* aliased to *E*_2,3_∗*E* and *υ*_23_∗*G*_12_. Since factors *E*_2,3_ and *E* have been significant, so probably the main reason for significance of *E*_1_∗*K* is due to the interaction of these two significant factors (*E*_2,3_∗*E*); but for more confidence, factors *E*_1_ and *K* are kept for further investigation due to their higher effect (0.0126, 0.0131, resp.) comparing to *υ*_23_ and *G*_12_ (0.0121, 0.0091, resp.). Moreover, this result is in agreement with research of Haut Donahue et al. [9] that showed the importance of *E*_1_ and *K* for contact variables of the tibial plateau. Furthermore, the importance of axial/radial modulus (*E*_2,3_) and circumferential moduli of meniscus (*E*_1_), as a result of the present study, is consistent with the findings of Yao et al. [15], who revealed that the meniscal motion and deformation are most sensitive to the circumferential and radial/axial moduli of menisci.

**Figure 5:**
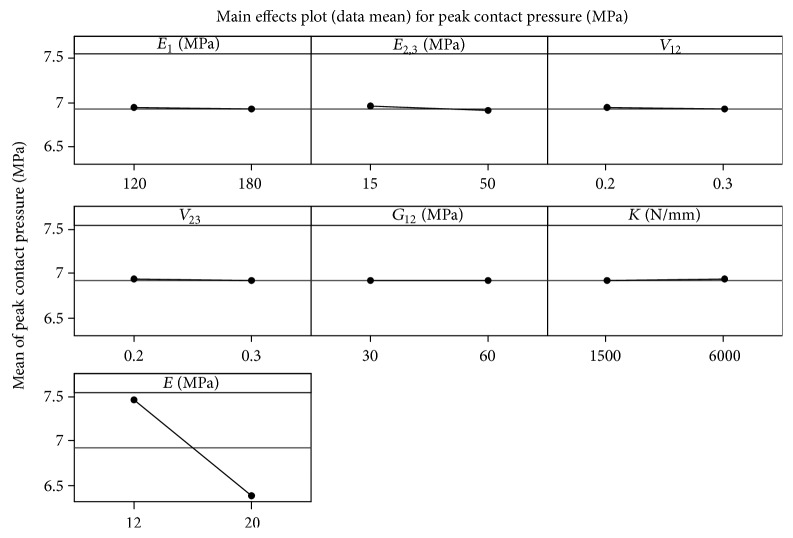
The main effects plot (data means) of peak contact pressure.

**Figure 6:**
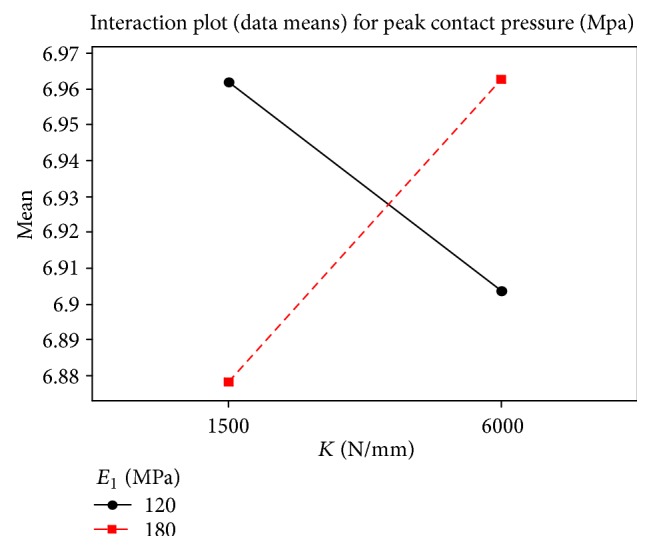
Interaction plot of peak contact pressure for *E*_1_ and *K*.

According to screening experiments, the following results can be estimated. (1)  Peak contact pressure for *E* = 12 (Mpa) is, on average, 1.1049 (Mpa) more than that for *E* = 20 (Mpa). (2) Peak contact pressure at *E*_2,3_ = 15 (Mpa) is, on average, 0.0496 (Mpa) more than that at *E*_2,3_ = 50 (Mpa). (3) The effect of 0.0714 for *E*_1_∗*K* can be interpreted to mean that the effect of combining the high level factor *E*_1_ with *K* (*E*_1_ = 180 Mpa, *K* = 1500, and 6000 N/mm) is, on average, 0.0714 more than the effect of low level of factor *E*_1_ with *K* (*E*_1_ = 120 Mpa, *K* = 1500, and 6000 N/mm). (4) However, the effect of other factors and their interaction are not significant.  Figure 5 shows the main effect of factors. The contact pressure decreases by increasing the *E*, *E*_1_, *E*_2,3_, *υ*_12_, and *υ*_23_ and increases by increasing the *G*_12_ and *K*. It is obvious that the main effect of factor *E* is much larger than the other factors and overshadows them. Interaction effect of *E*_1_ and *K* is shown in Figure 6. 

From the results of the screening experiment, factors of *E*, *E*_1_, *K*, and *E*_2,3_ were collected for more investigation by response surface method (RSM). 

### 3.2. Response Surface Method 

 In this section, the response surface method was used as a statistical design of experiment tool, in order to produce precise maps based on mathematical models leading to optimum performance [41]. The regression model was built in two phases. First started with linear model but due to the lack of linear fit, quadratic model was applied subsequently. For choosing the level of factors which screened out in the last section, less interactions with other factors were considered (*υ*_23_ = 0.2, *G*_12_ = 60 MPa, and *υ*_12_ = 0.2). The Box-Behnken method was used at three levels of each factor and a single center point was considered because the FEM experiments include no actual experimental error; thus, duplication of center point was not necessary [42, 43]. The results of simulation runs and Box-Behnken design in RSM are given in Table 4. In this step, in order to assess more levels of each factor, other ranges of variables were chosen. 

Estimated linear regression for peak contact pressure (PCP) is as follows:

(1) $$\begin{array}{c} \text{PCP}=8.80182-0.13871*E^{***}\;\left(R_{\text{adj}}^{2}=95.8\%\right). \end{array}$$

Results of ANOVA revealed that with 99% confidence, increase of one unit of *E* (*P* value <0.01) will result in decrease of peak contact pressure by 0.13871 MPa.

**Figure 7:**
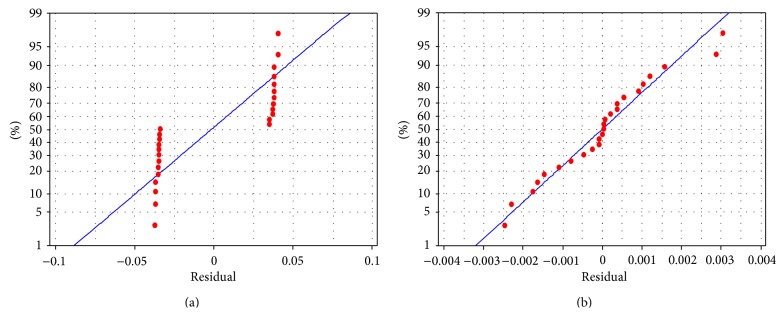
Normal probability plot of residuals. (a) Linear and (b) full quadratic regression model (response is peak contact pressure).

Although, the adjusted *R* ^2^ demonstrates that 95.8% of variation in peak contact pressure can be explained by variation in *E*, Figure 7(a) shows that the residuals of linear regression model for peak contact pressure are not normal; hence, the regression model should be revised. The full quadratic regression coefficients for peak contact pressure are estimated as follows:

(2) PCP=13.4733−0.7265∗E∗∗∗+0.0182∗E2∗∗∗+0.0001∗E∗E2,3∗∗ (Radj2=100%).

**Figure 8:**
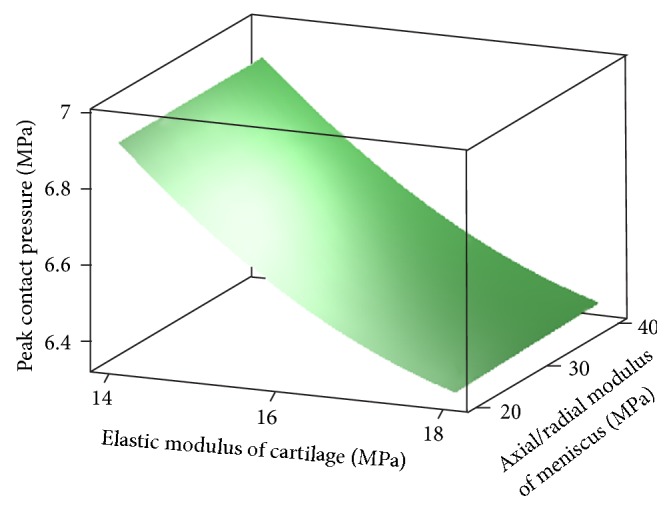
Surface plot of peak contact pressure versus *E*_2,3_ and *E* (at *E*_1_ = 1500 (Mpa), *K* = 4000 (N/mm)).

**Figure 9:**
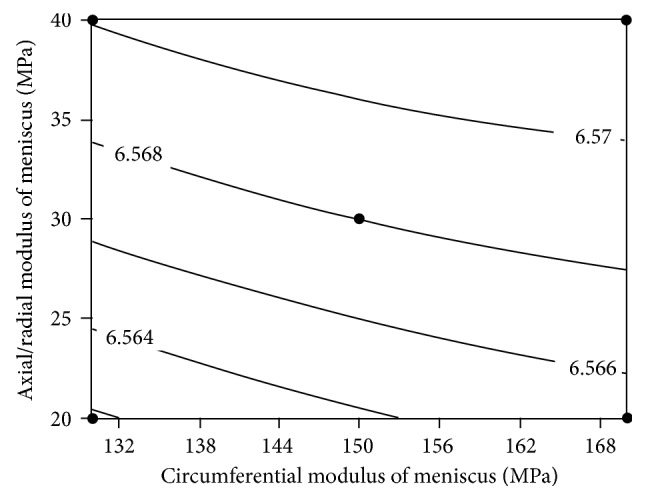
Contour plot of peak contact pressure (Mpa) versus *E*_2,3_, *E*_1_ (at *E* = 16 (Mpa), *K* = 4000 (N/mm)).

**Figure 10:**
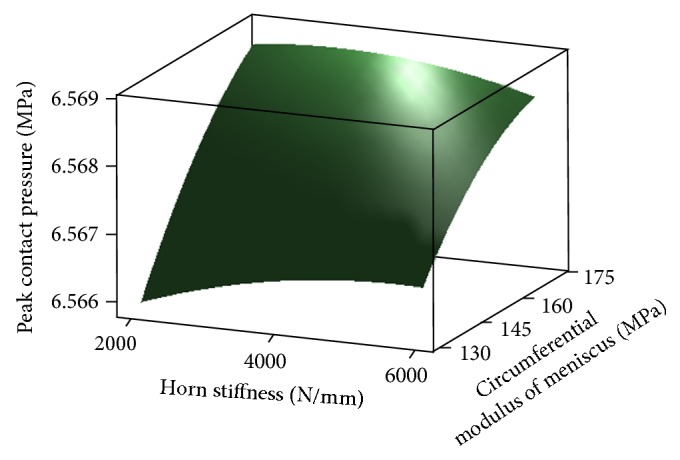
Surface plot of peak contact pressure versus *E*_1_ and *K* (at *E* = 16 (Mpa), *E*_2,3_ = 30 (N/mm)).

According to the outputs of ANOVA the following can be concluded. (1)  *E* affects peak contact pressure with 99% confidence (∗∗∗means *P* value <0.01), as −0.7265∗*E* + 0.0182∗*E* ^2^, and with 95% confidence (∗∗means *P* value <0.05) as 0.0001  *E*∗*E*_2,3_, if the effects of factor *E*_2,3_ are held constant. (2)  *E*_2,3_ affects the peak contact pressure with 95% confidence (*P* value <0.05) as 0.0001∗*E*∗*E*_2,3_, if the effects of factor *E* are held constant. (3) There is no reason to believe the importance, of other factors, interactions and any other quadratic effects. Residuals of full quadratic regression model are shown in Figure 7(b). According to the results of RSM, surface plot of peak contact pressure versus *E*_2,3_ and *E* is shown in Figure 8. It demonstrates the negative effect of *E* on peak contact pressure. Contour plot of peak contact pressure in Figure 9 shows that *E*_2,3_ has more effect on contact pressure than *E*_1_.  Figure 10 represents that the peak contact pressure does not change when *K* is greater than 4000 (N/mm).

### 3.3. Optimization

**Figure 11:**
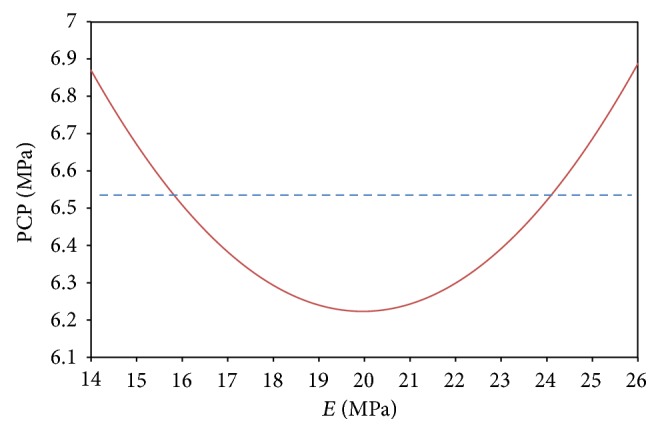
Behavior of quadratic estimated model for peak contact pressure with respect to *E*.

The optimal region to run a process is typically determined after a sequence of experiments and developing empirical models. From a mathematical viewpoint, the objective is to find the operating conditions that maximize, minimize, or close the system's response to the true one. Therefore, the goal of this section is minimizing difference between estimated quadratic model obtained in the last section and experimental data of Brown and Shaw [31]. By considering 99% confidence, the estimated quadratic model will only include factor *E*.  Figure 11 shows the behavior of peak contact pressure with respect to *E*. In regression analysis, usually developing a model includes the fewest numbers of explanatory variables which permit an adequate interpretation:

(3) Min  f=PCP−6.5Min  f=(13.4733−0.7265∗E+0.0182∗E2)−6.5   s.t  14≤E≤18.

The value of *E* was obtained to be 16.059 (Mpa) by solving the above nonlinear problem using generalized reduced gradient (GRG) algorithm on Microsoft Excel software. Furthermore, according to Figure 11, it is obvious that the minimum contact pressure is at *E* = 20 (MPa).

 Further analyses were carried out after optimizing the *E* and removing its strong shadow on the other factors.  Table 5 shows the Box-Behnken design with one center point, three factors, and peak contact pressure. In this stage, wider ranges of parameters were considered to investigate the maximum effects of factors.

Estimated full quadratic regression coefficients for peak contact pressure by considering optimized value of *E*, factors *E*_1_, *E*_2,3_, and *K* are as follows:

(4) PCP=6.517+0.00099∗E2,3+0.00019∗E1−0.00001∗E2,32 (Radj2=99.8%).

**Figure 12:**
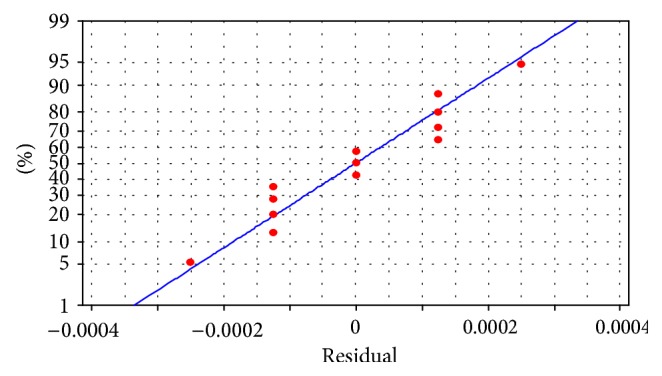
Normal probability plot of residuals for regression model using optimized value of *E*.

**Figure 13:**
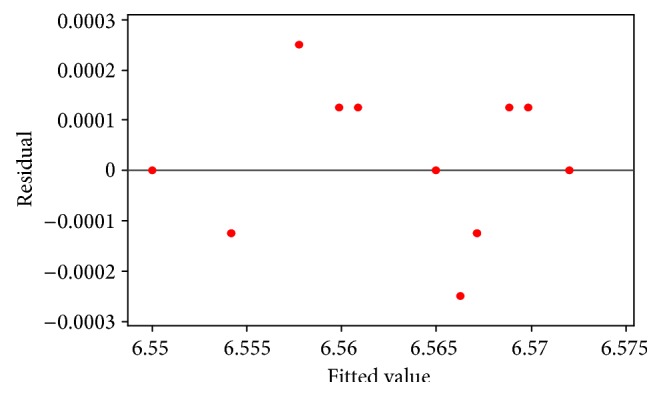
Residuals versus the fitted values in estimated regression model using optimized value of *E*.

According to the outputs of ANOVA, it can be concluded that with 99% confidence, *E*_2,3_ affects peak contact pressure as 0.00099∗*E*_2,3_ − 0.00001∗*E*_2,3_^2^, *E*_1_ as 0.00019∗*E*_1_ and there is no reason to believe the importance of factor *K*, other interactions, and any other quadratic effect.  Figure 12 shows normality of residual in the above estimated regression model and Figure 13 demonstrates random distribution of residuals versus the fitted values.

In order to find the optimum value for *E*_1_ and *E*_2,3_, it was tried to minimize the difference between the above estimated quadratic model and experimental data again:

(5) Min  f=PCP−6.5Min  f=(−0.00001∗E2,326.517+0.00099∗E2,3Min  f   +0.00019∗E1−0.00001∗E2,32)−6.5s.t  100≤E1≤200     20≤E2,3≤60.

The optimum values for *E*_1_ and *E*_2,3_ were 100 and 20 MPa, respectively. The suitable value for *K* can be considered ≥2000 N/mm. This value supports the idea that meniscal replacement surgery should attach the horns through a technique providing high stiffness. Using the values obtained from the optimization process, finally the results of the proposed model were tested by running another two simulations with two compressive loads of 1000 N [30] and 3000 N [31] at 0 degrees of flexion. The errors between FEA and experimental data of peak contact pressure decreased to be less than 10% and 5% for 1000 and 3000 N, respectively. 

It should be pointed out that in the FEM, some anatomical geometries are missing or simplified depending on the complexity of the problem. Ligaments were not included in our FE model. The posterior cruciate ligament (PCL) and lateral collateral ligament (LCL) are slack under axial compressive loading at 0 flexion angle [8], but the anterior cruciate ligament (ACL) and the medial collateral ligament (MCL) both contribute in the axial compression experienced by the joint. Under no external load, the joint is primarily compressed due to the prestress in these two ligaments and the axial compression sustained by the joint is, thus, greater than the applied external load. Therefore, the influence of missing these two ligaments in this FEA is that the results of peak contact pressure only correspond to the external load. Future studies will consider including ACL and MCL. However, according to Haut Donahue et al. [9], contact characteristics are not sensitive to the nonlinear material properties of MCL during axial compression. Furthermore, cartilage can be assumed to have linear elastic material property in contact analyses from the biphasic solution, but the huge influence of its elastic modulus on contact pressure might indicate the requirement for more precise material model in this component. This is supported by the study of Julkunen et al. [14] which showed that the mechanical responses of the cartilage under different loading conditions are dependent on tissue composition and structure. Therefore, future investigations will focus on the effect of anisotropic nonlinear behavior of cartilage on contact outputs. Moreover, it will be interesting to investigate the sensitivity of contact pressure to material properties under different degrees of flexion, which was not considered in this research. 

## 4. Conclusion 

A sensitivity analysis of tibiofemoral peak contact pressure to the material properties of soft tissue was performed and design of experiments methods was used to reduce the number of program runs and to minimize the contact pressure error. The present study evaluated the effect of cartilage elastic modulus and interaction effects of the parameters in addition to previous research. It was demonstrated that elastic modulus of the cartilage is the most influential factor. Another important finding was that after cartilage elastic modulus, interaction of axial/radial modulus with elastic modulus of cartilage, circumferential and axial/radial moduli of meniscus are significant factors. The importance of circumferential and axial/radial moduli of meniscus as a result of this study is in agreement with the past predictions. Furthermore, this research demonstrated the complex relations between material properties of tissue and contact pressure of tibiofemoral joint. The result of sensitivity analyses can be used as a guideline for experimental efforts intended at determining material properties of soft tissue, because estimating the most sensitive parameters should be done precisely. However, this analysis is only valid under full extension loading mode and with elastic assumptions of soft tissues. Further biomaterial studies may reveal more factors or more realistic form of material properties of human tissues. However, more investigation in this regard based on DOE techniques will provide a remarkably versatile strategy for analysis of knee joint biomechanics and help researchers with faster and more reliable analysis.

## Figures and Tables

**Table 1:** Name and variation range of factors.

Factor	Name	Range of variation	References
A	*E*_1_ (Mpa): circumferential modulus of meniscus	100–200	[20, 21, 23]
B	*E*_2,3_ (Mpa): axial/radial modulus of meniscus	15–60	[20, 23, 24]
C	*υ*_12_: in-plane Poisson's ratio	0.1–0.4	[9]
D	*υ*_23_: out-of-plane Poisson's ratio	0.1–0.35	[9]
E	*G*_12_ (Mpa): shear modulus of meniscus	27.7–77.7	[9]
F	*K* (N/mm): stiffness of meniscus horn attachment	500–30,000	[9]
G	*E* (Mpa): elastic modulus of cartilage	5–20	[25–28]

**Table 2:** Actual values of 2^7-3^ screening design and response.

Run no.	*E*_1_ (Mpa)	*E*_2,3_ (Mpa)	*υ*_12_	*υ*_23_	*G*_12_ (Mpa)	*K* (N/mm)	*E* (Mpa)	Peak contact pressure (Mpa)
1	180	50	0.2	0.3	30	1500	12	7.396
2	120	50	0.3	0.2	30	1500	20	6.381
3	120	15	0.2	0.2	30	1500	12	7.546
4	120	15	0.2	0.3	30	6000	20	6.361
5	180	15	0.3	0.2	30	6000	12	7.542
6	180	50	0.3	0.3	60	6000	20	6.389
7	120	15	0.3	0.3	60	1500	12	7.537
8	180	50	0.2	0.2	30	6000	20	6.386
9	180	15	0.3	0.3	30	1500	20	6.364
10	180	15	0.2	0.2	60	1500	20	6.366
11	120	50	0.3	0.3	30	6000	12	7.400
12	180	15	0.2	0.3	60	6000	12	7.533
13	120	50	0.2	0.2	60	6000	12	7.492
14	180	50	0.3	0.2	60	1500	12	7.386
15	120	50	0.2	0.3	60	1500	20	6.384
16	120	15	0.3	0.2	60	6000	20	6.362

**Table 3:** Considered terms, effects, and alias structure in 1/8 fractional design.

Term	Effect	Alias structure (up to order 3)
*E*_1_	−0.0126	*E*_1_ + *E*_2,3_∗*V*_12_∗*G*_12_ + *E*_2,3_∗*K*∗*E* + *V*_12_∗*V*_23_∗*E* + *V*_23_∗*G*_12_∗*K*
*E*_2,3_	−0.0496	*E*_2,3_ + *E*_1_∗*V*_12_∗*G*_12_ + *E*_1_∗*K*∗*E* + *V*_12_∗*V*_23_∗*K* + *V*_23_∗*G*_12_∗*E*
*V*_12_	−0.0129	*V*_12_ + *E*_1_∗*E*_2,3_∗*G*_12_ + *E*_1_∗*V*_23_∗*E* + *E*_2,3_∗*V*_23_∗*K* + *G*_12_∗*K*∗*E*
*V*_23_	−0.0121	*V*_23_ + *E*_1_∗*V*_12_∗*E* + *E*_1_∗*G*_12_∗*K* + *E*_2,3_∗*V*_12_∗*K* + *E*_2,3_∗*G*_12_∗*E*
*G*_12_	0.0091	*G*_12_ + *E*_1_∗*E*_2,3_∗*V*_12_ + *E*_1_∗*V*_23_∗*K* + *E*_2,3_∗*V*_23_∗*E* + *V*_12_∗*K*∗*E*
*K *	0.0131	*K* + *E*_1_∗*E*_2,3_∗*E* + *E*_1_∗*V*_23_∗*G*_12_ + *E*_2,3_∗*V*_12_∗*V*_23_ + *V*_12_∗*G*_12_∗*E*
*E *	−1.1049	*E* + *E*_1_∗*E*_2,3_∗*K* + *E*_1_∗*V*_12_∗*V*_23_ + *E*_2,3_∗*V*_23_∗*G*_12_ + *V*_12_∗*G*_12_∗*K*
*E*_1_∗*E*_2,3_	−0.0124	*E*_1_∗*E*_2,3_ + *V*_12_∗*G*_12_ + *K*∗*E*
*E*_1_∗*V*_12_	0.0129	*E*_1_∗*V*_12_ + *E*_2,3_∗*G*_12_ + *V*_23_∗*E*
*E*_1_∗*V*_23_	0.0126	*E*_1_∗*V*_23_ + *V*_12_∗*E* + *G*_12_∗*K*
*E*_1_∗*G*_12_	−0.0126	*E*_1_∗*G*_12_ + *E*_2,3_∗*V*_12_ + *V*_23_∗*K*
**E**_1_∗**K**	**0.0714**	**E**_1_∗**K** + **E**_2,3_∗**E** + **V**_23_∗**G**_12_
*E*_1_∗*E*	0.0169	*E*_1_∗*E* + *E*_2,3_∗*K* + *V*_12_∗*V*_23_
*E*_2,3_∗*V*_23_	−0.0069	*E*_2,3_∗*V*_23_ + *V*_12_∗*K* + *G*_12_∗*E*
*E*_1_∗*E*_2,3_∗*V*_23_	0.0129	*E*_1_∗*E*_2,3_∗*V*_23_ + *E*_1_∗*V*_12_∗*K* + *E*_1_∗*G*_12_∗*E* + *E*_2,3_∗*V*_12_∗*E* + *E*_2,3_∗*G*_12_∗*K* + *V*_12_∗*V*_23_∗*G*_12_ + *V*_23_∗*K*∗*E*

The bold item shows the most important term.

**Table 4:** Response, factors, and levels for the Box-Behnken experimental design.

Run no.	*E* (Mpa)	*E*_1_ (Mpa)	*K* (N/mm)	*E*_2,3_ (Mpa)	Peak contact pressure (Mpa)
1	16	150	2000	40	6.572
2	14	130	4000	30	6.917
3	16	150	2000	20	6.562
4	16	150	6000	40	6.572
5	16	130	4000	40	6.571
6	14	150	2000	30	6.918
7	18	150	6000	30	6.363
8	14	150	4000	40	6.916
9	16	150	6000	20	6.562
10	18	150	4000	40	6.367
11	16	150	4000	30	6.568
12	16	170	6000	30	6.569
13	18	150	2000	30	6.363
14	14	150	6000	30	6.918
15	18	130	4000	30	6.362
16	18	170	4000	30	6.364
17	16	130	4000	20	6.560
18	18	150	4000	20	6.359
19	16	170	2000	30	6.569
20	14	150	4000	20	6.920
21	16	130	6000	30	6.567
22	14	170	4000	30	6.918
23	16	130	2000	30	6.566
24	16	170	4000	40	6.573
25	16	170	4000	20	6.564

**Table 5:** Box-Behnken experimental design after optimizing the *E*.

Run no.	*E*_1_ (Mpa)	*E*_2,3_ (Mpa)	*K* (N/mm)	Peak contact pressure (Mpa)
1	100	60	6000	6.566
2	200	40	10000	6.567
3	150	20	2000	6.554
4	150	40	6000	6.565
5	150	60	2000	6.569
6	200	20	6000	6.558
7	100	40	10000	6.561
8	200	60	6000	6.572
9	150	60	10000	6.570
10	200	40	2000	6.567
11	100	40	2000	6.560
12	150	20	10000	6.554
13	100	20	6000	6.550

*G*_12_ = 60 (MPa), *υ*_23_ = 0.2, *υ*_12_ = 0.2, and *E* = 16.059 (MPa).
